# The mitochondrial genome of the springtail *Bourletiella arvalis* (Symphypleona, Collembola)

**DOI:** 10.1080/23802359.2019.1664347

**Published:** 2019-09-12

**Authors:** Chiara Leo, Francesco Nardi, Francesco Frati, Pietro Paolo Fanciulli, Claudio Cucini, Matteo Vitale, Claudia Brunetti, Antonio Carapelli

**Affiliations:** Department of Life Sciences, University of Siena, Siena, Italy

**Keywords:** Basal hexapods, springtails, mitogenomics, Collembola, springtail phylogeny

## Abstract

The complete mitochondrial genome of the springtail *Bourletiella arvalis* (Fitch, 1863) is herein described and applied to a Bayesian phylogenetic analysis, inclusive of all the Collembola mitochondrial DNAs sequenced so far. The gene content and order, as well as the nucleotide composition, conform with the well-known features of hexapods’ mitochondrial genomes. The phylogenetic analysis supports the monophyly of Collembola, Poduromorpha, Entomobryomorpha and Symphypleona. However, no mtDNA from Neelipleona is available to date, therefore limiting the application of mitochondrial genomes to further investigate springtail systematics.

Specimens of the springtail *Bourletiella arvalis* were sampled at Monsindoli, nearby Siena (Collection site: Siena, Italy, 43°16.73988′N, 11°19.34442′E). Whole genomic DNA was extracted from four different samples (Voucher specimen IDs: BOU_1, BOU_2, BOU_3, BOU_4, preserved at Life Sciences Department of University of Siena). The mitochondrial DNA was amplified and sequenced as described in Carapelli et al. (2019).

The complete mitochondrial genome (mtDNA) of *B. arvalis* was assembled using the software Sequencher 4.4.2 (Gene Codes Corporation, Ann Arbor, MI, USA). The consensus sequence was submitted to the tRNA secondary structure prediction tool ARWEN (Laslett and Canbäck 2008). The software identified 21 out of 22 tRNAs, missing the *trnL (uag)*, whose secondary structure was manually derived from the genome sequence; the tRNA genes were then mapped on the contig. The mitochondrial protein-coding genes (PCGs) were detected searching for their start and stop codons, following all the possible reading frames and with a direct comparison with already annotated springtail mtDNAs. The ribosomal DNA genes were identified by manually deriving the secondary structures of the 5′- and 3′- end domains, following Gillespie et al. (2006). The annotated genome was deposited in GenBank under the accession number: NC_039558.

The *B. arvalis* mtDNA is a circular molecule of 14,794 bp in length. It contains the common 37 genes, arranged along the chromosome in the order considered ancestral for Pancrustacea (Boore et al. 1998). Two non-coding regions were detected: the A + T-rich region, involved in the regulation of replication and transcription processes (long 256 bp), and a smaller spacer of 140 bp between the *trnF* and *nad5*. Ten PCGs show the canonical start codon for Methionine (ATG/ATA), whereas the codon for Isoleucine (ATT) is used in *nad1*, *nad4L*, and *nad5* genes. Most of the PCGs (7/13) show truncated stop codons (TA–/T––), presumably post-transcriptionally restored (Lavrov 2007). As generally observed among arthropods, the mtDNA of *B. arvalis* is strongly biased toward a higher content of A and T bases (A = 39.6%, T = 33.4%, C = 12.9%, G = 14.1%).

The *B. arvalis* PCGs were aligned with those of the 14 mtDNAs of springtails available on GenBank, plus three outgroup species (list in the caption of Figure 1). The alignment was performed using the online software RevTrans 1.4 (Wernersson and Pedersen 2003) and the ambiguous aligned positions were removed from the data set through the GBlock tool (Castresana 2000). The final data set was tested with the software PartitionFinder 2.1.1 (Lanfear et al. 2016). The model selected (GTR + I + Γ) was applied to a Bayesian phylogenetic analysis using the software MrBayes 3.2 (Ronquist et al. 2012), run with four chains for 10^6^ generations, with a sampling of one tree/1000 iterations and 25% of burn-in.

**Figure 1.**
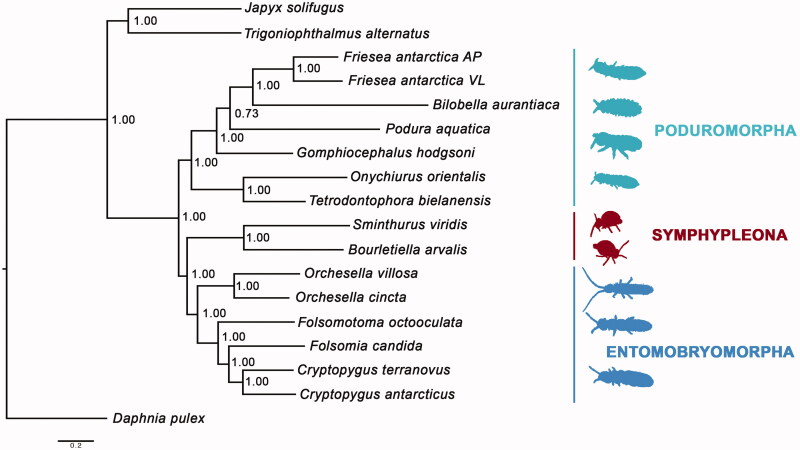
Bayesian phylogenetic tree inferred applying the 13 mitochondrial protein-coding genes of the following three outgroup and springtail species: *Daphnia pulex* (NC000844), *Trigoniophthalmus alternatus* (NC010532), *Japyx solifugus* (NC007214), *Friesea antarctica* from Antarctic Peninsula (AP; NC010535), *Friesea antarctica* from Victoria Land (VL; EU124719), *Bilobella aurantiaca* (NC011195), *Podura aquatica* (NC006075), *Gomphiocephalus hodgsoni* (NC005438), *Onychiurus orientalis* (NC006074), *Tetrodontophora bielanensis* (NC002735), *Sminthurus viridis* (NC010536), *Bourletiella arvalis* (NC039558), *Orchesella villosa* (NC010534), *Orchesella cincta* (NC032283), *Folsomotoma octooculata* (NC024155), *Folsomia candida* (KU198392), *Cryptopygus terranovus* (NC037610), *Cryptopygus antarcticus* (NC010533). At each node, the posterior probability values are shown.

The phylogenetic tree obtained recovered Collembola and the springtail orders monophyletic (Figure 1). Traditionally, Poduromorpha and Entomobryomorpha were clustered together; instead, in the present analysis, Entomobryomorpha was sister group of the globular-shaped Symphypleona (Figure 1). However, no definitive conclusion can be stated on the evolutionary relationships of springtail orders since no mtDNA is available for Neelipleona.
